# Supplementary figures and images for: Adjuvant Activity of Synthetic Lipid A of Alcaligenes, a Gut-Associated Lymphoid Tissue-Resident Commensal Bacterium, to Augment Antigen-Specific IgG and Th17 Responses in Systemic Vaccine
Source: Vaccines (Basel). 2020 Jul 20;8(3):395. doi: 10.3390/vaccines8030395 (PMC7565795; doi:10.3390/vaccines8030395)

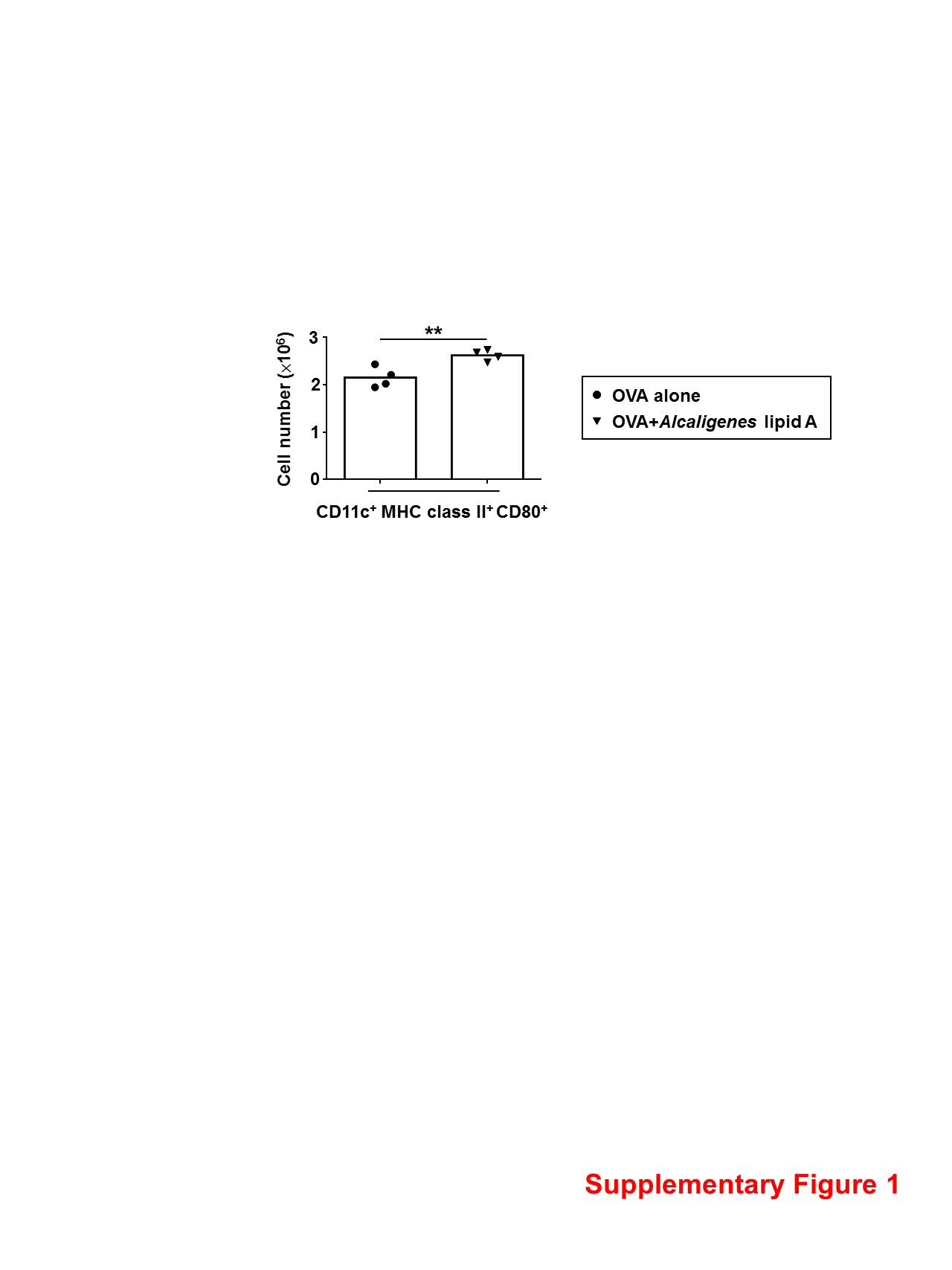

Supplement: Supplementary file 1 [file vaccines-08-00395-s001.zip › Supplementary Figure 1.TIF]

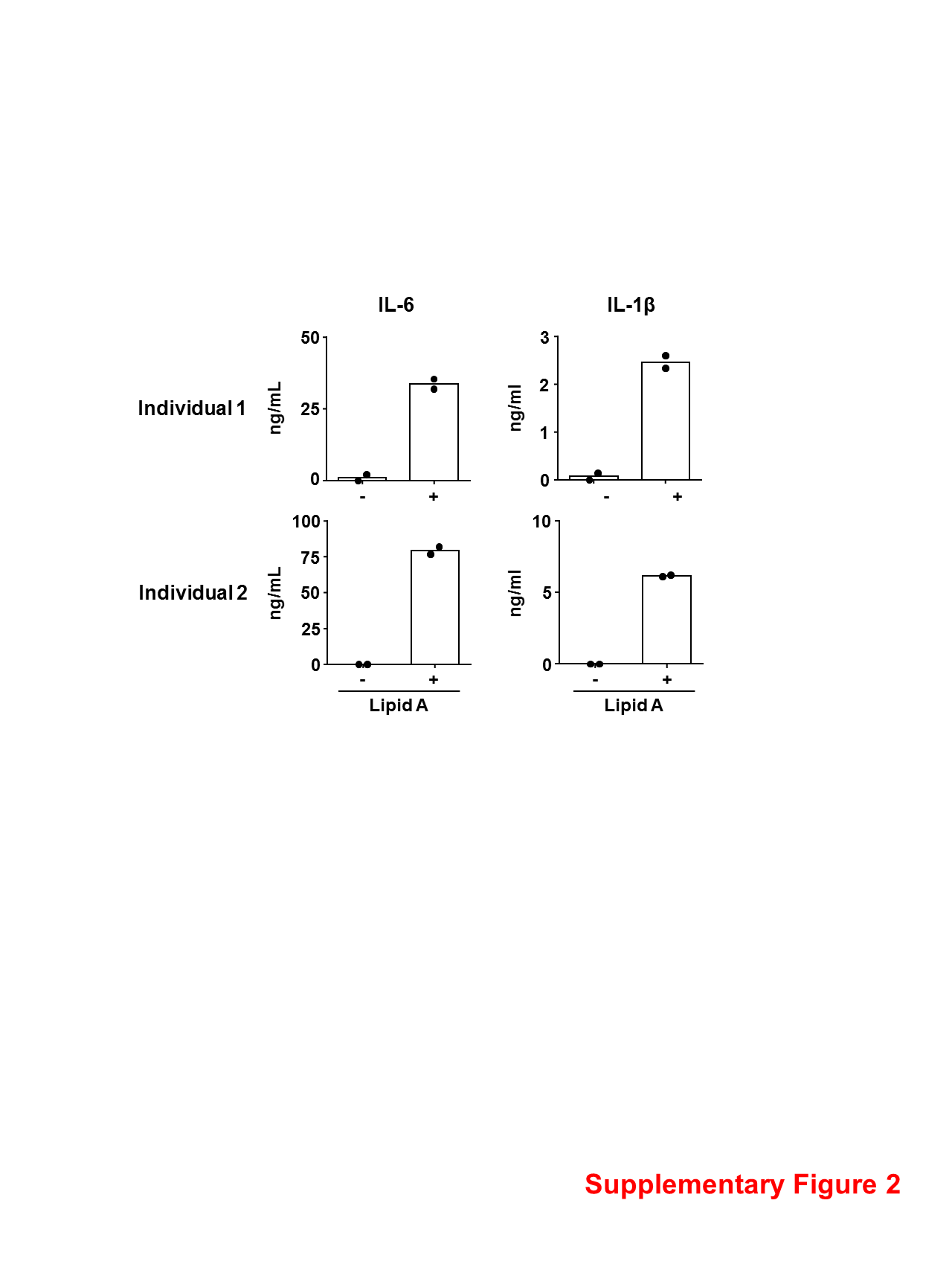

Supplement: Supplementary file 1 [file vaccines-08-00395-s001.zip › Supplementary Figure 2.TIF]
